# Angiogenesis modulated by CD93 and its natural ligands IGFBP7 and MMRN2: a new target to facilitate solid tumor therapy by vasculature normalization

**DOI:** 10.1186/s12935-023-03044-z

**Published:** 2023-09-02

**Authors:** Yang Li, Lei Fu, Baokang Wu, Xingqi Guo, Yu Shi, Chao Lv, Yang Yu, Yizhou Zhang, Zhiyun Liang, Chongli Zhong, Shukun Han, Feng Xu, Yu Tian

**Affiliations:** 1 Department of General Surgery, Shengjing Hospital of China Medical University, No.36.Sanhao stress, Heping District, Shenyang, 110004 Liaoning Province China; 2 Department of Surgery, The First Affiliated Hospital of Jinzhou Medical University, Jinzhou, 121001 Liaoning Province China

**Keywords:** CD93, IGFBP7, MMRN2, Vasculature normalization, Immune therapy

## Abstract

The tumor vasculature was different from the normal vasculature in both function and morphology, which caused hypoxia in the tumor microenvironment (TME). Previous anti-angiogenesis therapy had led to a modest improvement in cancer immunotherapy. However, antiangiogenic therapy only benefitted a few patients and caused many side effects. Therefore, there was still a need to develop a new approach to affect tumor vasculature formation. The CD93 receptor expressed on the surface of vascular endothelial cells (ECs) and its natural ligands, MMRN2 and IGFBP7, were now considered potential targets in the antiangiogenic treatment because recent studies had reported that anti-CD93 could normalize the tumor vasculature without impacting normal blood vessels. Here, we reviewed recent studies on the role of CD93, IGFBP7, and MMRN2 in angiogenesis. We focused on revealing the interaction between IGFBP7-CD93 and MMRN2-CD93 and the signaling cascaded impacted by CD93, IGFBP7, and MMRN2 during the angiogenesis process. We also reviewed retrospective studies on CD93, IGFBP7, and MMRN2 expression and their relationship with clinical factors. In conclusion, CD93 was a promising target for normalizing the tumor vasculature.

## Introduction

Vascularization was a critical process in both normal and tumor tissue [1]. Abnormality and dysfunction of the vasculature in solid tumors caused hypoxia in the TME, while hypoxia stimulated vascular endothelial growth within tumors, which in turn limited immune cell infiltration and reduced the effect of immunotherapy drugs [2, 3]. Moreover, oxidative stress could impair endothelial function, inducing inflammation and facilitating adhesion by circulating cancer cells [4, 5].

CD93 had been identified as one of the top 20 genes in tumor angiogenesis [6]. Many studies had shown that loss of CD93, either using siRNA knockdown of CD93 in ECs or investigating angiogenesis or tumor development in CD93 knockout mice, was associated with disruption of endothelial junctions and increased vascular permeability [7, 8]. However, recently, Sun et al. showed that the IGFBP7-CD93 axis was related to disordered tumor vasculature, and a CD93-neutralizing antibody could enhance immune response, associated with vascular normalization, increased endothelial activation, and increased leukocyte recruitment [9]. The discrepancy between the result of the deficiency and the antibody-targeting models might be because the loss of CD93 in CD93^−/−^ mice was complete, while the ablation of CD93 by antibody blockade was transient and relatively incomplete. Moreover, CD93 expression was upregulated in tumor vasculature, and antibody blockade might inhibit the overexpressed CD93 in tumor vessels and normalize the CD93 signal. [9].

Sun and colleagues also reported that overexpression of the IGFBP7-CD93 pathway in patients who received anti-PD-1/PD-L1 treatment was related to worse treatment effect, and blocking this axis by CD93-neutralizing antibody could promote tumor therapy by regulating the TME [9]. Interestingly, CD93 monoclonal antibody (mAb) blockade could specifically improve the vascular function in the TME while having no effect on the normal vasculature in healthy organs. In contrast, anti-VEGFR mAb markedly influenced normal vessel homeostasis. Moreover, anti-VEGFR but not CD93 blockade influenced angiogenesis during wound healing. [9]. Therefore, IGFBP7-CD93 axis inhibition might be a promising and safer therapeutic target than VEGF for normalizing the tumor vasculature.

## The CD93 receptor

CD93 (also known as C1qr, C1qRp, and AA4) was a heavily glycosylated protein belonging to the C-type lectin domain (CTLD) group 14 family [10]. The O-glycosylation was critical for maintaining CD93 expression on the membrane surface [11]. CD93 was previously named C1qr because it was thought to be the receptor of C1q, but a subsequent study demonstrated that C1q and C1qr do not mediate their biological functions by direct interactions [12, 13].

CD93 consisted of an N-terminal signal peptide, a CTLD (D1), a sushi-like domain, five EGF-like domain repeats (D2), a mucin-like region (D3), a single-pass transmembrane region (D4), and a cytoplasmic tail (D5) [14–16]. Expression of CD93 was identified in different cell types, such as monocytes, neutrophils, platelets, microglia, and vascular ECs [10, 12, 17]. Numerous scRNA-seq studies now prove to us several endothelial phenotypes co-exist [18]. In this review, we focus on CD93 which expresses on vascular ECs.CD93 expression was high during tumor neovascularization but low in quiescent blood vessels [6, 16, 19].

In addition to its expression on the cell surface, many stimuli, such as inflammation, could cause CD93 ectodomain cleavage from the membrane surface to form soluble CD93 (sCD93), and the sCD93 was identified in culture supernatant or human blood samples. [20, 21]. Researchers had demonstrated that sCD93 contains an N-terminal carbohydrate recognition domain and EGFR domain repeats that could play a proangiogenic function in EC via the EGF-like domain [20, 22]. The plasma concentration of sCD93 was regarded as a biomarker for specific diseased and physiological processes such as allergic asthma [23], systemic sclerosis [24], coronary artery disease [25], and glucometabolic regulation [26]. In this review, we focus on CD93 that expresses on angiogenic tip ECs, and the tumor angiogenesis process that was impacted by CD93.

## The natural ligands of CD93

To date, the CD93 receptor had been demonstrated that had two natural ligands, IGFBP7 and MMRN2, bind to the different domains of CD93 receptor [9, 16, 27].

IGFBP7 (also known as IGFBP-rP1, AGM, T1A12, TAF, mac25, and PSF) was a glycoprotein of approximately 30 kDa that belongs to the IGFBP family [28]. IGFBP7 expression was upregulated during physiological and pathological angiogenesis processes, such as brain injury and tumor neovascularization [27, 29–31]. p53 could activate IGFBP7-AS1 transcriptional expression, and IGFBP7-AS1 could increase IGFBP7 mRNA stability to regulate IGFBP7 expression [32]. In addition to IGFBP7-AS1, the participation of Smarcb1 was required for IGFBP7 transcriptional activation [33]. In the tumor vasculature, hypoxia, VEGF, and TGF-β1 were associated with IGFBP7 expression in vascular ECs [9, 34, 35].

MMRN2 (also known as Multimerin-2 or Endoglyx-1) was a member of the family of elastin microfibrillar interface proteins (EMILINs) [36]. Unlike CD93 and IGFBP7, during the angiogenesis process, the angiogenic stimulatory factors such as VEGF-A could potentially cause MMRN2 mRNA downregulation [37]. MMRN2 stabilized CD93 localization to endothelial filopodia by inhibiting proteolysis [8].

DX domain was defined as a 79-amino acid domain localized between D1 and D2 domains [10]. MMRN2-CD93 interaction precisely involved a region overlapping the D1 and DX domains (D1X fragment) in CD93 and coiled-coil domains in MMRN2 [16, 38]. CD93 interacted with MMRN2 by the DX domain mainly, while the D1 domain was required to maintain the DX domain fold properly because the D1 domain was characterized by two β-sheets organized in anti-parallel β-strands spaced by two helixes, whereas the DX domain exhibited lower structural stability, characterized by the absence of secondary structure elements [16].

MMRN2-CD93 played a role in promoting specific steps of angiogenesis processes, including forming angiogenic sprouts and enabling angiogenic sprouts, fibronectin interactions, and fibrogenesis to occur. [8, 16, 38, 39]During tumor vascularization, the formation of the MMRN2-CD93 complex ensured that focal adhesion kinase (FAK) phosphorylation, β1 integrin activation, and fibronectin fibrillogenesis in ECs [8]. Blockade of the MMRN2-CD93 interaction could impact endothelial cell adhesion, migration, and angiogenesis [16], and knockdown of either MMRN2 or CD93 caused apparent deformation of the fibronectin fibrillar network formation in vitro [8].

## The effects of CD93, IGFBP7, and MMRN2 on the vasculature

### CD93

**Fig. 1:**
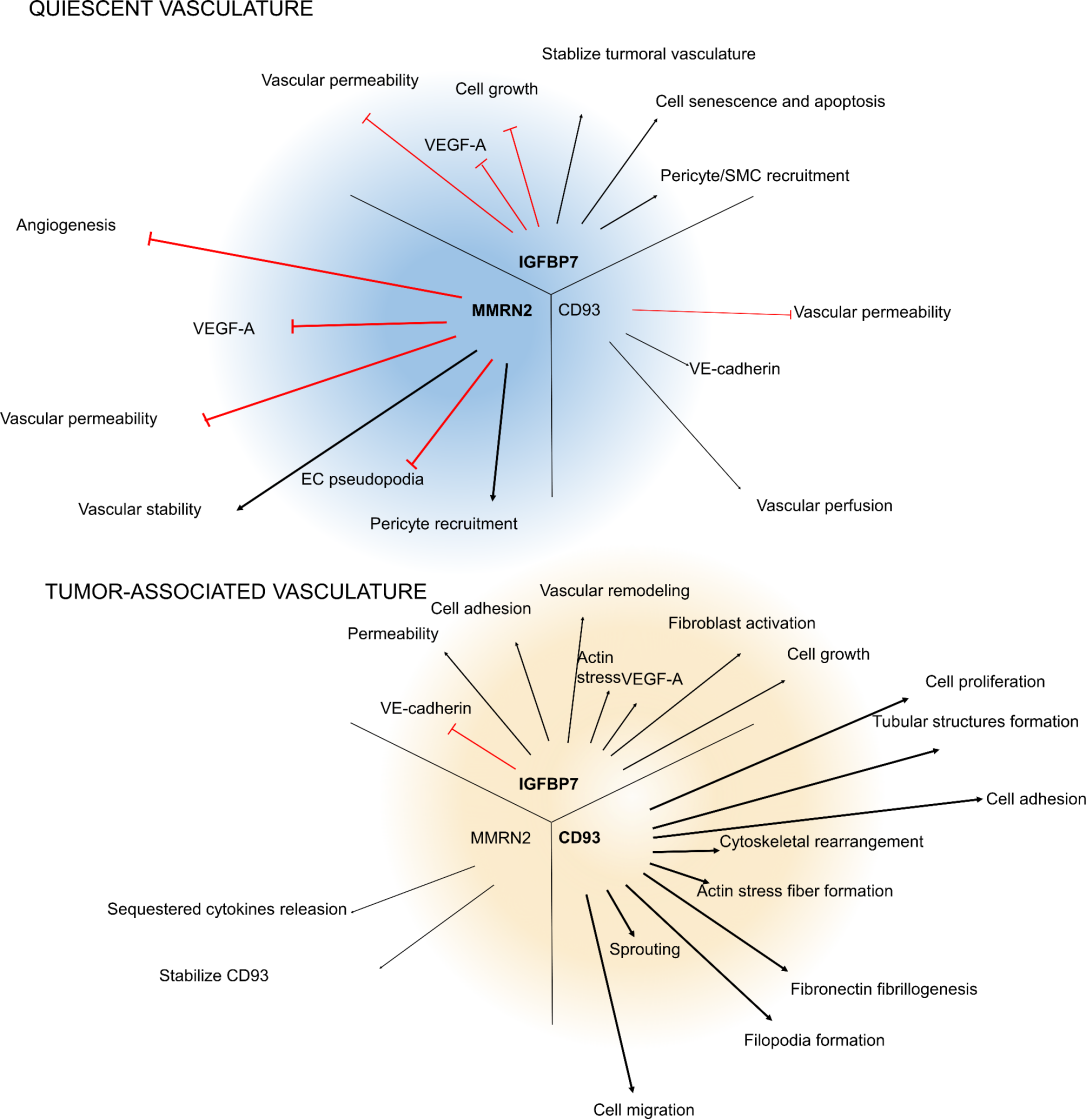
Effects of CD93, IGFBP7, and MMRN2 on the vasculature were listed in the figure Alt Text: The figure is divided into two small figures, quiescent vasculature, and tumor-related vasculature, and lists the roles of related molecules in shape similar to a dandelion

CD93 participated in cell proliferation, migration, sprouting, and the formation of tubular structures in EC, which were critical steps in angiogenesis [7, 10, 40]. (Fig. 1)

One of the mechanisms by which CD93 impacted angiogenesis was influencing VE-cadherin. It had been demonstrated that downregulation of CD93 would lead to disruption of VE-cadherin in endothelial junctions and then increased vascular permeability [7, 10, 12]. Moreover, by analyzing the developing mouse retina vasculature, Roberta and colleagues demonstrated that CD93 and α5β1 integrins showed colocalization in endothelial filopodia, and CD93 could regulate β1 integrin signaling and fibronectin fibrillogenesis as well as promote the formation of filopodia in ECs [8].

### IGFBP7

IGFBP7 could exert both synergistic and antagonistic functions with VEGF-A. On the one hand, IGFBP7 synergized with VEGF-A to partially regulate vascular remodeling [29]. Using morpholino oligomers to block IGFBP7 partly impacted the proangiogenic effect of VEGF-A, such as vascular sprouting and remodeling [29]. By analyzing the cytoskeletal change in IGFBP7-treated HUVECs and two-well chamber assay, Eriko et al. reported that IGFBP7 affected the permeability of blood vessels by impacting actin stress fibers and loosening intercellular junctions mediated by VE-cadherin in ECs [34]. Despite IGFBP7 stimulating neither the growth nor migration of ECs [34], it obviously stimulated normal fibroblast proliferation, migration, and induced fibronectin expression [27].

On the other hand, IGFBP7 could also serve as an angiogenesis inhibitor to antagonize VEGF-A-mediated hyperpermeability and tube formation in the vasculature [41–44]. Some literature also reported that IGFBP7 could also exert tumor inhibition function. IGFBP7 promoted smooth muscle cell (SMC) or pericyte recruitment and differentiation to inhibit tumor cell growth, promote cell senescence and stabilize the tumor vasculature [44–47], and tumor growth could be promoted by IGFBP7 knockdown in vivo also was reported in another literature, which was consistent with the result mentioned above [48]. The contrary effect might occur because of different forms of IGFBP7 expression, including its expression on tumor cells and its expression in the extracellular matrix (ECM). When IGFBP7 was expressed by tumor cells, it played a role in promoting malignant mesenchymal cell and EMT-phenotype epithelial cell growth in an anchorage-independent manner, which contrasts with its IGFBP7 tumor inhibition function in ECM [49]. Epithelial-mesenchymal transition (EMT) is a known cellular program that is critical for embryogenesis, wound healing, and malignant progression. In the context of tumors, EMT confers cancer cells with increased tumor initiation and metastatic potential, as well as greater resistance to the elimination of several treatment options [50].

**Fig. 2:**
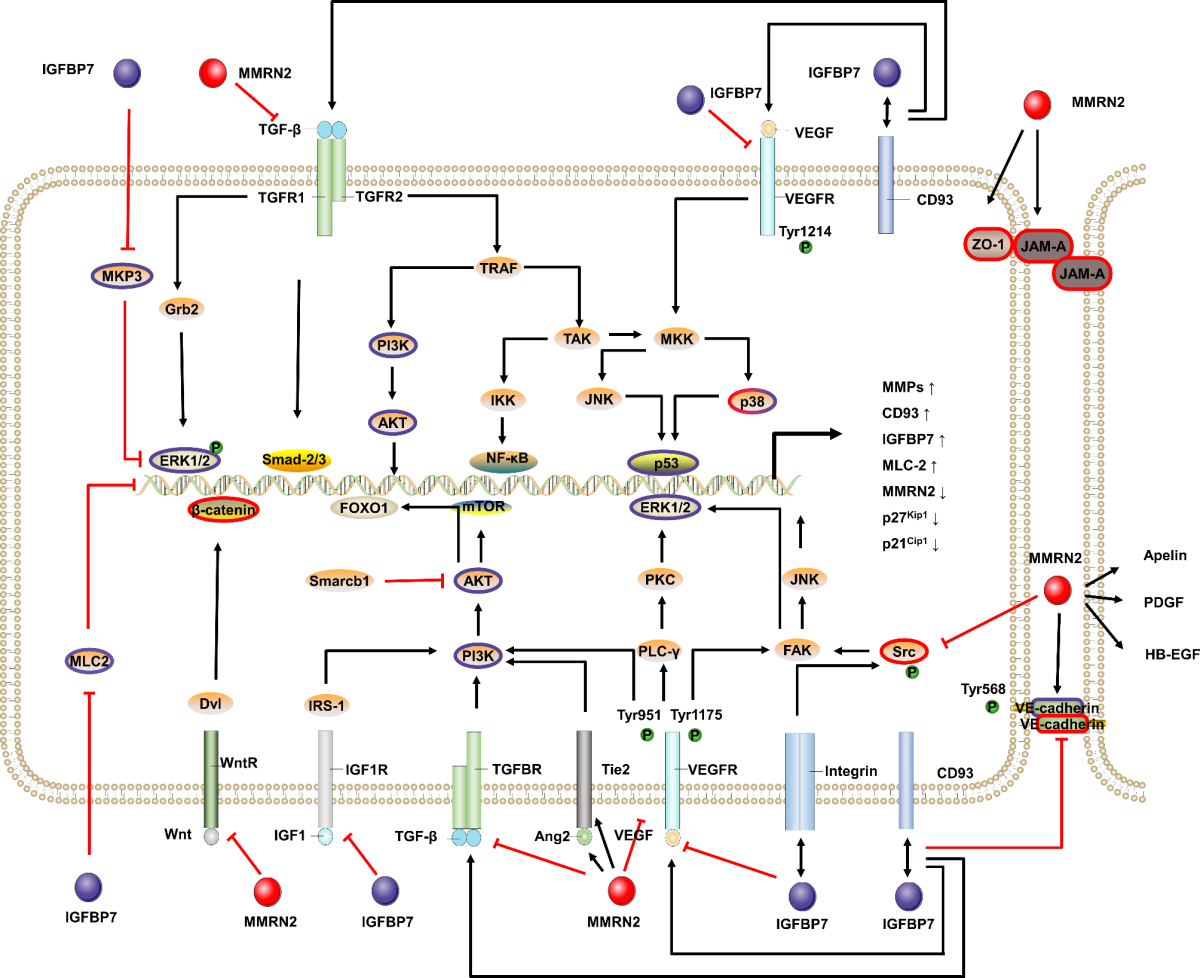
The signal molecules modulated by IGFBP7 and MMRN2. The red icons represent the signal molecule regulated by MMRN2, and the purple icons represent the signaling molecules regulated by IGFBP7. 1). IGFBP7 promoted TGF-1R and AKT phosphorylation and activated the p38MAPK pathway, upregulating p53, p27Kip1, and p21Cip1. 2). IGFBP7 inhibited AKT and MPK3 activity, MEK and ERK1/2 phosphorylation, and COX-2 and VEGF mRNA expression. IGFBP7 interrupts the IGF1R-IGF1/2 interaction and, in turn, inhibited the downstream PI3K-AKT pathway. 3) TGF-β1 induced by TGF-β1/ALK5/Smad-2 and VEGF induced IGFBP7 expression. MKP3 could inhibit IGFBP7 expression. IGFBP7 transcriptional activation required Smarcb1 participation, while Smarcb1 inhibited AKT activation. 4) MMRN2 inhibited p38 activation and Src, VEGFR2, and VE-cadherin phosphorylation. 5) MMRN2 downregulated VEGFR1, VEGFR2, Tie2, MLC2, Apelin, and Ang-2. 6) MMRN2 upregulated VE-cadherin, β-catenin, ZO-1, and JAM-A, MMRN2 upregulated some cytokines, such as Ang2, PDGF, and HB-EGF, and 7) VEGFF-A could downregulate MMRN2. Alt Text: There is a connected cell in the figure in which the intrinsic pathways involved in the relevant molecules are marked

Mechanically, firstly, IGFBP7 expression upregulation was related to the inhibition of activation of the p38-MAPK pathway; upregulation of p53, p27Kip1, and p21Cip1; G1/S cell cycle arrest; senescence induction; and phosphorylation-mediated activation and activity of AKT (Fig. 2) [48, 51, 52]. Next, IGFBP7 competitively interfered with the interaction between IGF1R and IGF1/2, causing inactive IGF1R accumulation and downstream PI3K–AKT signal inhibition [53, 54]. However, another study reported that IGFBP7 knockdown inhibited TGF-1R and AKT phosphorylation in tyrosine kinase inhibitor (TKI)-resistant cells. [55]. Then, IGFBP7 inhibited the phosphorylation of MEK and ERK1/2 in human umbilical vein endothelial cells (HUVECs) [42], which was consistent with the result in another study reporting that IGFBP7 mutation caused BRAF/MEK/ERK pathway upregulation in patients with familial retinal artery microaneurysms [56]. Next, IGFBP7 knockdown could increase the MKP3 level, while MKP3 knockdown increased IGFBP7 expression levels [57]. Finally, by examining the effect of IGFBP7 on VEGF-induced PGE2 production, Kazuhiro et al. found that IGFBP7 could inhibit COX-2 and VEGF mRNA expression, and its influence could be reversed after the knockdown of IGFBP7 [42].

Following the discovery that IGFBP7 was the ligand of CD93 and promoted EC angiogenesis via CD93 [9], most signal molecules involved in the VEGF and TGF-β signal axis were impacted by IGFBP7 reviewed above, were speculated by IGFBP7-CD93 interaction (Fig. 2). Nevertheless, the particular signal pathway still needed further to be confirmed.

### MMRN2

MMRN2 might act as a homeostatic barrier to inhibit EC migration, angiogenesis, and tumor growth, promote vascular maturation, and maintain vascular stability [37, 58–61]. Moreover, by generating MMRN2^−/−^ mice, Rosanna et al. demonstrated that the abnormal vasculature in MMRN2^−/−^ mice caused hypoxia in the tumor vessels and decreased responsiveness to chemotherapy, which was consistent with the importance of MMRN2 in proper vessel homeostasis and stabilization maintenance [60]. Therefore, MMRN2 expression might influence the function of the tumor vasculature and, in turn, influence its effect on cancer therapy [37].

By forming a network on the membrane around ECs [37], MMRN2 could affect the TME by binding to many types of cytokines [61]. For example, by binding to VEGF-A via carbohydrate chains, MMRN2 caused VEGF to sequester from receptors to inhibit the VEGF/VEGFR2 pathway [58, 61], while the VEGF-A might be realized after MMRN2 degradation [37].

MMRN2 deposition also played a critical role during EC-pericyte crosstalk. Pericyte and EC interactions promoted MMRN2 expression, while MMRN2 acted as an adhesion molecule between pericytes and ECs and provided a docking site for pericytes to play a critical role in connecting ECs and pericytes [59]. MMRN2 plays a role in the recruitment of pericytes through the stimulation of PDGF and HB-EGF expression in ECs [59]. When pericytes were recruited to the abluminal surface of vascular guidance tunnels formed by ECs, it promoted vascular maturation, vascular basement membrane matrix deposition, fibronectin, nidogen perlecan, and laminin isoform induction [59, 62].

Mechanically, MMRN2 inhibited VEGFR2 Tyr1175 and Tyr1214 phosphorylation and p38 activation [52, 58, 61]. (Fig. 2) In addition, MMRN2 knockdown was related to increased VEGFR2 Tyr951, VE-cadherin Tyr568, and Src phosphorylation [60]. MMRN2^−/−^ mice could develop a vasculature, but they had defects in cell-cell junctions caused by the increased phosphorylation levels of VEGFR2 Tyr949, which was the counterpart of Tyr951 in humans, along with defects in pericyte recruitment and vascular permeability. ECs that lack MMRN2 present defects in VE-cadherin, β-catenin, ZO-1, JAM-A, and circumferential actin bundled and increased MLC2 [60]. Moreover, MMRN2 could also upregulate Ang-2, Tie-2, and downregulate Apelin [59].

### Signal cascades involved in CD93

**Fig. 3:**
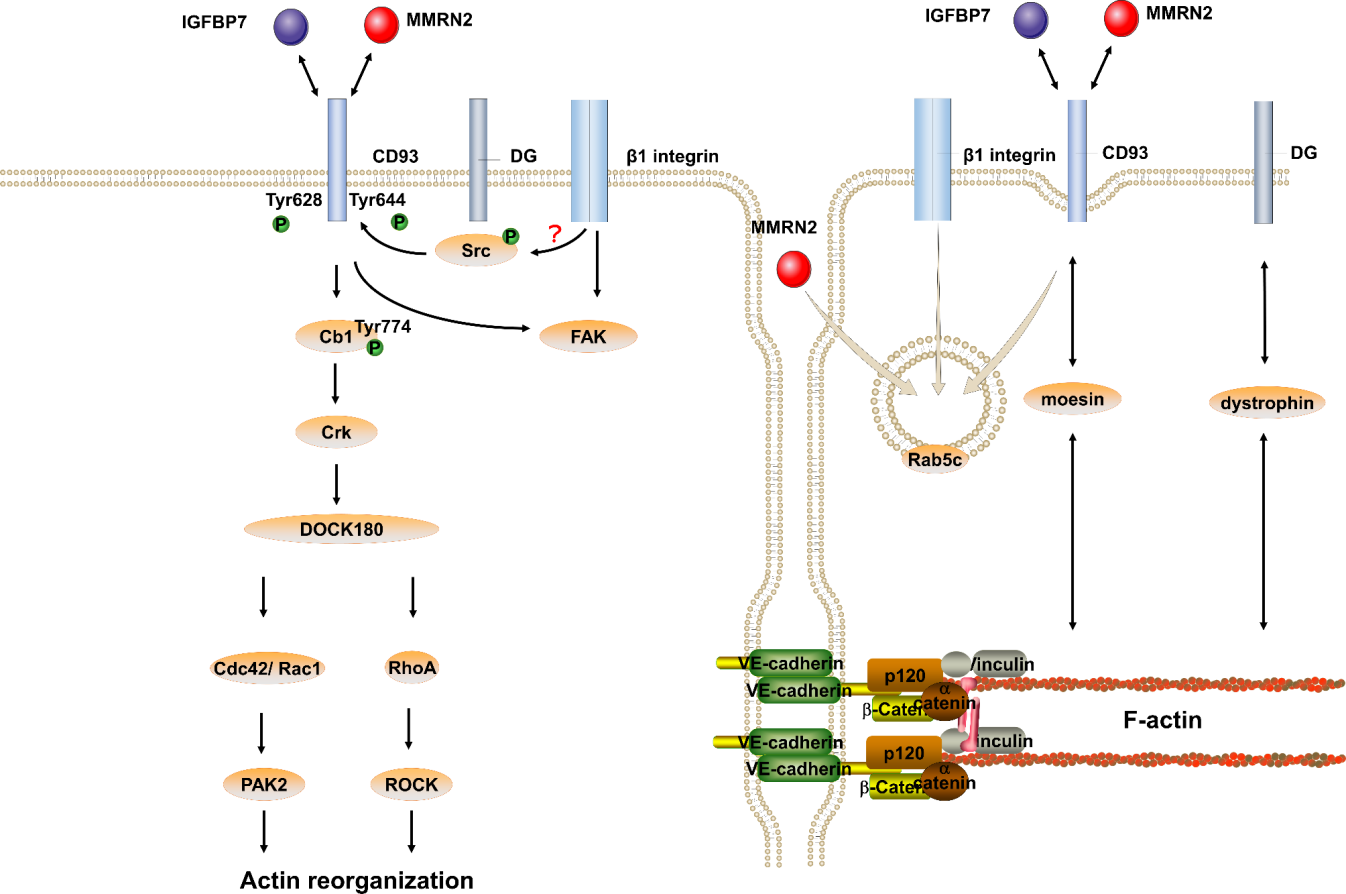
The signal pathway mediated by CD93 in ECs. (1) Integrin participated in the activation of Src and FAK, and CD93 ensured FAK activation. (2) Cell adhesion on laminin caused DG phosphorylation, phosphorylated DG recruits Src, integrin-induced Scr activation, and activated Src was induced to phosphorylate the CD93 cytoplasmic domain. Phosphorylated CD93 recruits and phosphorylated Cb1, phosphorylated the Cb1 receptor, and interacted with Crk, which interacted with DOCK180 and regulated downstream Rho GTPases, including Rac1, Cdc42, and RhoA. (3) The cytoplasmic region of CD93 interacted with moesin and F-actin. (4) Dystroglycan interacted with dystrophin, and dystrophin interacted with F-actin. (5) The small GTPase Rab5c participated in cycling CD93 to the surface of ECs, and CD93, MMRN2, and β1 integrin form a complex in the Rab5c endosomal compartment Alt Text: There are two connected cells in the figure, the left side depicts the intrinsic signaling pathway, and the right side depicts the changes in the related protein backbone and the transport of molecules

CD93, Cbl, Crk, Rac1, Cdc42, and RhoA were involved in cytoskeletal remodeling (Fig. 3) [63, 64], which participated in EC adhesion and migration. When actin stress fiber formation was downregulated in ECs, cell adhesion, and cell contact were influenced [7, 10, 65]. Integrins were proteins that played a vital role in linking the cytoskeleton and signaling pathways [66], and they participated in the activation of many signaling proteins, such as FAK, Src, and Cas [67]. In vascular ECs, IGFBP7 was also the ligand of integrin αvβ3. Moreover, IGFBP7 and integrin αvβ3 were upregulated in the tumor vasculature [34].

Dystroglycan (DG), a laminin-binding protein that could be upregulated in tumor vascular ECs, played a critical role in angiogenesis and had close interactions with CD93. CD93 and DG could promote endothelial cell migration and form tubular structures [68]. The intracellular part of β-dystroglycan interacted with dystrophin, and dystrophin interacted with the actin cytoskeleton [69]. The cytoplasmic region of CD93, by interacting with moesin and F-actin, played a critical role in retrieving CD93 within adhering and migrating cells. The endosomal downregulation of CD93 restrained CD93 localization to the migration leading edge [70]. Moreover, the small GTPase Rab5c played a critical role in cycling CD93 to the surface of ECs. CD93, MMRN2, and β1 integrin form a complex in the Rab5c endosomal compartment. Complex cycling to the lateral surface of ECs occurs during clathrin-independent endocytosis [70].

When the cell interacted with the stroma, integrin provided the signal to induce Src activation [65], which might participate in Src activation in the process of CD93 phosphorylation. After cell adhesion on laminin caused phosphorylation of DG, which had been demonstrated that it also expressed in ECs under a dynamic regulation and might participate in angiogenesis [71], phosphorylated DG recruits Src and Tyr628 and Tyr644 on the cytoplasmic domain of CD93 were phosphorylated by Src [65, 68]. Phosphorylated CD93 recruits Cbl and caused Cbl phosphorylation on Tyr774, providing a site for Crk to bind [65, 68]. Phosphorylated Cb1 recruits Crk, and Crk interacted with DOCK180 to regulate Rho GTPase activity. Rho GTPases, including Rho, Rac, and Cdc42, were well known to play a vital role in cell migration [63, 65, 72, 73]. By the pathway mentioned above, CD93 regulated the activity of Rho, Rac, and Cdc42 at the cell migration edge, increasing Rac1 and Cdc42 activity while decreasing RhoA activity specifically [63, 64]. Besides impacting the signaling in ECs, CD93 also transmits signals to β-catenin, and β-catenin transmitted signals to activate Zfp503 expression. Zfp503 bind to the promoter of Gfap with the assistance of Grg5 to inhibit Gfap expression in neurons [74].

### The impact of CD93, IGFBP7, and MMRN2 on solid tumors

The expression of CD93, IGFBP7, and MMRN2 was altered in the TME. CD93 expression was upregulated in colorectal cancer and nasopharyngeal carcinoma. IGFBP7 expression was upregulated in breast cancer, glioblastoma, and esophageal adenocarcinoma and downregulated in colorectal cancer, colon cancer, glioma, hepatocellular carcinoma, high-grade serous ovarian carcinoma, pancreatic cancer, and thyroid carcinoma. Moreover, it had been demonstrated that IGFBP7 expression upregulation was associated with poor clinical outcomes in breast cancer, lung cancer, esophageal adenocarcinoma, and soft-tissue sarcomas (STS), was associated with good clinical outcomes in cholangiocarcinoma (CCA), hepatocellular carcinoma (HCC), glioma, high-grade serous ovarian carcinoma (HGSC), esophagogastric junction adenocarcinoma (EJA), and pancreatic cancer. However, discordance occurs in gastric cancer and colorectal cancer. This discordance might be due to the analysis of total tumor RNA expression versus IHC of tumors. Another source of discordance was the antibody used for the IHC. MMRN2 expression was upregulated in low-grade glioma and downregulated in gastric cancer, and MMRN2 expression upregulation was associated with poor clinical outcomes in low-grade glioma. CD93 expression upregulation was associated with poor clinical outcomes in glioblastoma and nasopharyngeal carcinoma (NPC). The relationships among CD93, IGFBP7, and MMRN2 expression and clinical parameters were listed in Table 1.

**Table 1:** CD93, IGFBP7, and MMRN2 expression and clinical parameters in various solid tumors

Human tumor type	molecule	sample	Expression level compared to normal tissue	The relationship between molecule expression and clinical parameters	Ref.
Breast cancer	IGFBP7	Tumor sample	Not done	Low IGFBP7 expression was related to less recurrence risk. with high IGFBP7 expression, the prognosis was dependent on host factors and treatment	[75]
	IGFBP7	Tumor sample	Evaluated	Not done	[34]
Colorectal cancer	IGFBP7	Serum sample	Evaluated	No significant differenced	[77]
	IGFBP7	Tumor sample	Not done	IGFBP7^+^ was related to a worse prognosis, including early recurrence and shorter survival time compared to IGFBP7^-^	[78]
	CD93	Tumor sample	Elevated	Not done	[80]
	CD93	Plasma sample	Reduced	Not done	[80]
	IGFBP7	Tumor sample	Reduced	Low IGFBP7 expression promoted metastasis in colon cancer	[79]
Cholangiocarcinoma (CCA)	IGFBP7	Tumor sample	Not done	Patients with IGFBP7 high expression had better overall survival than IGFBP7 low expression.	[47]
Gastric cancer	IGFBP7	Tumor sample	Evaluated	Evaluated IGFBP7 expression was related to short survival	[82]
	IGFBP7	Tumor sample	Not done	IGFBP7 expression upregulation was associated with poor disease-specific survival, advanced stages in tumors with lymph node metastasis, and distant metastasis and recurrence.	[83]
	IGFBP7	Tumor sample	Reduced	IGFBP7 expression downregulation was associated with unfavorable clinical outcome	[85]
	IGFBP7	Tumor sample	Reduced	IGFBP7 expression downregulation was associated with poor prognosis, including the depth of invasion, lymph node metastasis, and TNM stage.	[84]
	MMRN2	Tumor sample	Reduced	Not done	[86]
Glioma	MMRN2	Tumor sample	Evaluated	MMRN2 expression upregulation was associated with shorter OS in low-grade glioma (IGG)	[89]
	IGFBP7	Tumor sample	Reduced	IGFBP7 expression downregulation was associated with larger tumor size and shorter overall survival.	[87]
	IGFBP7	Tumor sample	Evaluated	Not done in glioblastoma	[88]
	CD93	Tumor sample	Not done	CD93 expression was related to reduced survival, higher tumor grade in glioblastoma	[7]
Hepatocellular carcinoma (HCC)	IGFBP7	Tumor sample	Reduced	IGFBP7 expression downregulation was associated with advanced tumor stage and grade.	[44]
	IGFBP7	Tumor sample	Not done	Downregulation was related to poorer prognosis, including shorter disease-free survival (DFS) and overall survival (OS) rate.	[90]
Lung cancer	IGFBP7	Tumor sample	Not done	IGFBP7^+^ was related to metastasis in non-small cell lung carcinoma	[92]
	IGFBP7	Serum sample	Not done	IGFBP7 expression was positively related to the clinical stage and lymphatic metastasis in lung adenocarcinoma	[94]
Esophagogastric junction adenocarcinoma (EJA)	IGFBP7	Serum sample	Evaluated	IGFBP7 expression upregulation was associated with longer OS	[81]
Nasopharyngeal carcinoma (NPC)	CD93	Tumor sample	Evaluated	CD93 expression upregulation was associated with higher T classification, N classification, distant metastasis, clinical stage, and poor prognosis	[95]
High-grade serous ovarian carcinoma (HGSC)	IGFBP7	Tumor sample	Reduced	IGFBP7 expression downregulation was associated with shorter overall survival.	[96]
Oesophageal adenocarcinoma	IGFBP7	Tumor sample	Elevated	IGFBP7 expression upregulation was associated with lower median survival and 5-year survival.	[97]
Oropharyngeal cancer	IGFBP7	Salivary sample	Elevated	Not done	[98]
Pancreatic cancer	IGFBP7	Tumor sample	Reduced	IGFBP7 expression downregulation was associated with poor OS	[99]
Soft-tissue sarcomas (STS)	IGFBP7	Tumor sample	Not done	IGFBP7^+^ was associated with elevated metastatic risk compared to IGFBP7^-^	[101]
	IGFBP7	Blood samples	Evaluated	Not done	[101]
Thyroid carcinoma	IGFBP7	Tumor sample	Reduced	Not done	[48]

### Breast cancer

Godina et al. analyzed 878 samples from breast cancer patients meeting the requirements. Data on IGFBP7 expression at the mRNA level from 809 patients were downloaded from the TCGA database. They reported that low IGFBP7 expression at both the protein and mRNA levels is related to less aggressive clinical characteristics, such as a low recurrence risk [75]. Komiya et al. collected 32 breast cancer tissue specimens. By immunohistochemistry (IHC), they proved that IGFBP7 expression in blood vessels is upregulated in ductal carcinoma in situ (DCIS) compared with normal tissue, while IGFBP7 expression in cancer-associated fibroblasts (CAFs) is more extensive in invasive carcinomas than in DCIS [34]. Kaya et al. investigated the methylation status in 61 samples of tumour and adjacent normal tissues from breast cancer patients. By methylation-specific PCR, they proved that IGFBP7 methylation is more prevalent in tumour tissue than in adjacent normal tissues (90% vs. 59%). Moreover, methylation is more frequent in invasive ductal carcinoma (IDC) than in invasive mixed carcinoma (IMC), but it is not related to protein expression or other clinical characteristics [76].

### Colorectal cancer (CRC)

Qiu et al. collected serum samples from 115 CRC patients and 107 healthy controls. By enzyme-linked immunosorbent assay (ELISA), they proved that IGFBP7 expression is upregulated in CRC serum compared with healthy samples [77]. Adachi collected 89 formalin-fixed colorectal cancer samples and 5 liver metastatic tumours for IHC analysis. They reported that IGFBP7 expression was identified in 37 samples. Moreover, IGFBP7^+^ patients have an early recurrence within 12 months after surgery and a shorter survival time, and a worse prognosis than IGFBP7- patients with colorectal cancer [78]. Li et al. enrolled 81 pairs of colon cancer and adjacent nontumour tissues in their study. By IHC and real-time quantitative reverse-transcriptase PCR (qRT-PCR), they found that substantial variations in IGFBP7 were identified in stage III and IV colon cancer compared to adjacent nontumour tissue. IGFBP7 expression was downregulated on the invasive front of liver metastatic colon tissues [79].

Olsen et al. analyzed 101 selected colorectal cancer samples. By ELISA, IHC, western blot, gene expression analysis, and real-time PCR, they found that CD93 expression is upregulated in tumour tissue compared to normal tissue, while soluble CD93 in plasma from cancer patients is downregulated compared to plasma from healthy patients [80].

### Cholangiocarcinoma (CCA)

Yue et al. enrolled 33 paraffin-embedded CCA samples in their study. By applying IHC, they found that IGFBP7 expression upregulation was related to better overall survival in patients with CCA [47].

### Oesophagogastric junction adenocarcinoma (EJA)

Liu et al. collected serum samples from 120 EJA patients and 88 healthy controls to examine IGFBP7 expression by ELISA. They reported that IGFBP7 expression was upregulated in EJA patient serum compared with serum from healthy patients [81].

### Gastric cancer (GC)

Zhao et al. collected gastric specimens and downloaded data from the TIMER, Oncomine, TCGA, and GEO databases. They reported that the IGFBP7 expression level is upregulated in GC and is related to tumour stage, tumour grade, tumour status, and Helicobacter pylori infection. Moreover, IGFBP7 expression upregulation and IGFBP7 methylation downregulation are related to shorter survival, and IGFBP7 can act as an independent prognostic factor for GC. Furthermore, IGFBP7 is positively associated with inflammation-related pathways, ECM, and it is associated with various infiltrating immune cells, especially tumour-associated macrophages [82]. Sato et al. collected 219 gastric cancer samples to examine IGFBP7 expression at the protein and mRNA levels by IHC and qRT-PCR, respectively. They reported that IGFBP7 expression upregulation was positively correlated with poor disease-specific survival, depth of invasion, lymph node metastasis, distant metastasis, recurrence, and pathological stage. In addition, IGFBP7 expression at the mRNA level was higher in advanced stages, in tumours with lymph node metastasis, and in those with distant metastasis and recurrence [83]. Liu et al. performed a retrospective study of 247 gastric cancer patients. By qRT-PCR to examine the IGFBP7 expression level, they identified IGFBP7 in 138 samples of gastric cancer and adjacent nontumour tissue. Moreover, IGFBP7 expression was downregulated in tumour tissue compared with adjacent nontumour tissue at both the mRNA and protein levels. Low IGFBP7 expression is related to a poor prognosis, including the depth of invasion, lymph node metastasis, and TNM stage [84]. Kim et al. collected 393 gastric cancer samples and examined them by qRT-PCR, IHC, western blot, and methylation-specific PCR for IGFBP7 expression and methylation levels. They reported that the IGFBP7 expression level could act as an independent prognostic factor. Moreover, increased gastric cancer cell growth, invasion, and migration were identified following IGFBP7 knockdown, and gastric cancer cell growth inhibition and apoptosis were identified following IGFBP7 upregulation. In addition, the IGFBP7 methylation level was negatively related to IGFBP7 expression in gastric cancer [85]. Andreuzzien et al. enrolled 51 patients who agreed to undergo pCLE endomicroscopy analyses. They found MMRN2 expression was high in normal mucosa but showed changes in many patients [86].

### Glioma

Tian et al. collected 120 glioma samples and 20 normal brain tissue samples and subjected them to real-time PCR to examine IGFBP7 mRNA. They reported that IGFBP7 expression at the mRNA level was downregulated in glioma compared to normal brain tissue. Moreover, the IGFBP7 expression level was inversely related to tumour size and positively related to overall survival [87]. However, Pen and colleagues reported that IGFBP7 expression is upregulated in ECs and the vascular abluminal basal lamina in glioblastoma multiforme (GBM) [88].

Langenkamp et al. collected 235 samples and analyzed them by IHC for vascular CD93 expression. They reported that the CD93 expression level in the vasculature was positively related to poor survival in high-grade astrocytic glioma patients, and in high-grade astrocytic glioma patients, vascular CD93 expression is related to worse survival [7]. Lugano et al. enrolled 163 glioma samples and 2 healthy controls. They reported that the MMRN2 expression level is associated with WHO grade in glioma, while MMRN2 expression is low in normal brain vessels. Moreover, high CD93 expression in glioma is related to high MMRN2 expression. CD93 expression is negatively related to overall survival in WHO grade III-IV astrocytoma patients [8]. Zhao et al. downloaded related information from the TCGA dataset and reported that MMRN2 expression is upregulated in low-grade glioma (IGG) [89].

### Hepatocellular carcinoma (HCC)

Chen et al. collected 104 HCC samples and 9 normal adjacent nontumour samples. By applying IHC, they reported that IGFBP7 expression is lower in HHC than in normal liver tissue, and 26% of HCC patients carry IGFBP7 genomic deletions. Moreover, IGFBP7 is negatively related to tumour grades and stages in HCC and can inhibit cell growth and promote senescence [44]. Tomimaru et al. collected 104 patients diagnosed with HCC. By qRT-PCR, they reported that IGFBP7 was identified in 67 of 104 HHC patients. Moreover, IGFBP7 downregulation is related to a worse prognosis, and IGFBP7 can be used to predict patient outcomes [90]. Li et al. collected 217 serum samples from 136 hepatocellular carcinoma (HCC) patients and 46 chronic hepatitis B (CHB) patients. By applying methylation-specific PCR, they found that the frequency of IGFBP7 methylation in HCC patients was higher than that in CHB patients. The frequency of IGFBP7 methylation was also higher in HCC patients with vascular invasion than in patients without vascular invasion [91].

### Lung cancer

Zhao et al. collected 97 paraffin-embedded samples from patients diagnosed with non-small-cell lung carcinoma (NSCLC). By IHC, they reported that IGFBP7 is related to metastasis and high lymphatic vascular density and may promote lymph angiogenesis [92]. Suzuki et al. collected 56 NSCLC samples to investigate the promotor methylation level. By RT-PCR, they reported that the hypermethylation frequency of IGFBP7 was identified in 54% of 56 primary NSCLCs, and hypermethylation is related to the IGFBP7 expression level and various clinical features [93].

Chen et al. collected 90 serum samples from 60 lung adenocarcinoma patients and 30 healthy controls. After applying independent sample mean t-tests to analyze the data, they found that the IGFBP7 expression level in patient serum with metastatic lung adenocarcinoma was higher than that in lung adenocarcinoma in situ [94].

### Nasopharyngeal carcinoma (NPC)

Bao et al. collected 65 samples from NPC patients to examine their CD93 expression levels. By IHC and western blot analysis, they found that CD93 expression is upregulated in NPC and is related to T classification, N classification, distant metastasis, clinical stage, and a poor prognosis [95].

### High-grade serous ovarian carcinoma (HGSC)

Bao et al. collected 175 HGSC samples to examine IGFBP7 expression at the protein level by IHC. They reported that most HGSC samples showed downregulated IGFBP7 expression or its total absence. There was a strong relationship between IGFBP7 expression upregulation and extended overall survival [96].

### Oesophageal adenocarcinoma

Smith et al. collected 65 oesophageal adenocarcinoma samples to examine IGFBP7 expression at the protein level by IHC. They reported that IGFBP7 expression was identified in 52% of patients, and it was related to lower median survival and 5-year survival. Furthermore, IGFBP7 promoter methylation level upregulation was common in Barrett’s oesophagus and oesophageal adenocarcinoma and was related to IGFBP7 knockdown [97].

### Oropharyngeal cancer

Cüneyt Asım Aral et al. collected salivary samples from 68 individuals. They confirmed that salivary IGFBP7 expression in oropharyngeal cancer samples was significantly higher than that in normal samples [98].

### Pancreatic cancer

An et al. enrolled 190 pancreatic ductal adenocarcinoma (PDAC) patients. By IHC, they found that IGFBP7 expression was downregulated in pancreatic cancer tissue compared to adjacent nontumour tissue. Moreover, IGFBP7 downregulation was related to poor overall survival (OS) [99].

### Prostate cancer

Sullivan et al. collected 64 prostate cancer samples and 92 nontumour tissue samples. By denaturing high-performance liquid chromatography and bisulfite sequencing, they examined the methylation level in tumour tissue and normal tissue. They reported that the IGFBP7 methylation frequency is higher in prostate cancer and high-grade prostatic intraepithelial neoplasia samples than in adjacent normal tissue and benign prostatic hyperplasia samples. Moreover, IGFBP7 promoter methylation is inversely related to IGFBP7 expression [100].

### Soft-tissue sarcomas (STS)

Maria Serena Benassi et al. collected 145 high-grade STS samples. By IHC, they found that IGFBP7 expression was higher in tumours with metastasis than in tumours without metastasis. IGFBP7 expression upregulation was related to an elevated metastatic risk. They also examined IGFBP7 expression in the serum of 59 patients and confirmed that IGFBP7 expression was upregulated in patient serum compared with healthy serum. Moreover, IGFBP7 expression upregulation was more significant in synovial sarcoma and liposarcoma than in other STS histotypes [101].

### Thyroid carcinoma

Zhang and his colleagues collected 112 paraffin-embedded thyroid tumour samples. By IHC, they found that IGFBP7 expression at the protein level is considerably downregulated in follicular thyroid cancer (FTC) and anaplastic thyroid cancer (ATC) compared to normal thyroid, benign thyroid adenoma, and classical papillary thyroid cancer (PTC) tissues [48].

### Concluding remarks and future perspectives

In this article, we reviewed recent studies on the function of CD93, IGFBP7, and MMRN2 in angiogenesis. MMRN2 and IGFBP7 were the ligands of CD93 and could bind to CD93 simultaneously. We showed that CD93 mainly played roles in the tumor-associated vasculature, MMRN2 mainly played roles in the quiescent vasculature, and IGFBP7 could play roles in both the quiescent and tumor-associated tumors vasculature. The contrary function of IGFBP7 might be because IGFBP7 could play different roles at different doses and/or in different expression manners. Moreover, we reviewed the signaling pathways regulated by IGFBP7 and MMRN2, which mainly involved TGF-β and VEGF signaling, which played a critical role in angiogenesis. We also review the signaling pathways mediated by CD93. Furthermore, we reviewed recent clinical research on CD93, IGFBP7, and MMRN2 expression levels and the relationships between their expression levels, clinical prognosis, and pathological factors.

Abnormality vasculature in TME had restricted the drug delivery, then restricted the antitumor drugs’ therapy effect. CD93 might act as a promising target in vasculature normalization. In future research, many results need further investigation and verification. First, the therapeutic effect of targeting the IGFBP7-CD93 axis should be further investigated. Second, the mechanism by which IGFBP7 played its opposite roles should be further investigated. Third, the expression levels of CD93, IGFBP7, and MMRN2 in various solid tumors and their relationship with the clinical characteristics of tumor patients should be further investigated. Finally, the therapeutic effect of a combination of CD93 blockage and other antitumor drugs should be further investigated in solid tumors.

## Data Availability

Not applicable.

## Abbreviations

CCA: Cholangiocarcinoma
CTLD: C-type lectin domain
CRC: Colorectal cancer
ECs: Endothelial cells
ECM: Extracellular matrix
ELISA: Enzyme-linked immunosorbent assay
EJA: Esophagogastric junction adenocarcinoma
FAK: Focal adhesion kinase
GC: Gastric cancer
HCC: Hepatocellular carcinoma
HGSC: High-grade serous ovarian carcinoma
IGFBP7: Insulin-like growth factor binding protein 7
IHC: Immunohistochemistry
mAb: Monoclonal antibody
NPC: Nasopharyngeal carcinoma
SMC: Smooth muscle cell
STS: Soft-tissue sarcomas
TME: Tumor microenvironment
VEGF: Vascular endothelial-derived growth factor
qRT–PCR: Quantitative reverse-transcriptase PCR
